# Prevalence and determinants of antidepressant non-adherence among patients with major depressive disorder in Ethiopia: a multi-center cross sectional study

**DOI:** 10.1038/s41598-025-15102-9

**Published:** 2025-08-14

**Authors:** Gashaw Sisay Chanie, Eyayaw Ashete Belachew, Liknaw Workie Limenh, Alemante Tafese Beyna, Assefa Kebad Mengesha, Zemenu Wube Bayleyegn, Mihret Melese, Jember Azanaw, Ashenafi Kibret Sendekie, Wudneh Simegn

**Affiliations:** 1 Department of Clinical Pharmacy, School of Pharmacy, College of Medicine and Health Sciences, University of Gondar, Gondar, Ethiopia; 2 Department of Pharmaceutics, School of Pharmacy, College of Medicine and Health Sciences, University of Gondar, Gondar, Ethiopia; 3 Department of pharmacology, School of Pharmacy, College of Medicine and Health Sciences, University of Gondar, Gondar, Ethiopia; 4 Department of Social and Administrative Pharmacy, School of Pharmacy, College of Medicine and Health Sciences, University of Gondar, Gondar, Ethiopia; 5 Department of Environmental and Occupational Health and Safety, Institute of Public Health, College of Medicine and Health Sciences, University of Gondar, Gondar, Ethiopia; 6 Department of Human Physiology, School of Medicine, College of Medicine and Health Sciences, University of Gondar, Gondar, Ethiopia

**Keywords:** Antidepressant non-adherence, Adherence determinants, Major depressive disorder, Ethiopia, Psychiatric disorders, Adverse effects, Drug therapy

## Abstract

Non-adherence to antidepressant medication is a well-established factor contributing to treatment failure among patients with major depressive disorder. Addressing this issue is crucial not only for enhancing individual patient outcomes but also for alleviating the broader public health burden. The study aimed to assess antidepressant medication non-adherence and its determinants among patients with major depressive disorder at public hospital psychiatric clinics in Ethiopia. Between June 12, 2024, and November 13, 2024, a multicenter cross-sectional study was conducted at public hospital psychiatric clinics in Ethiopia. Antidepressant non-adherence was assessed using a self-reported tablet count tool with pharmacy refill records available in patients’ charts at the time of the interview. The severity of adverse drug reactions (ADRs) was evaluated using the Antidepressant Side-Effect Checklist (ASEC), while the Naranjo ADR Probability Scale was employed to determine the likelihood of ADRs. Data analysis was performed using SPSS version 26.0. Frequencies and percentages, were used to describe the characteristics of study participants. For factors associated with antidepressant non-adherence, multivariate logistic regression was conducted. The association between explanatory variable and non-adherence was assessed using odds ratios (ORs) with 95% CIs. The prevalence of antidepressant medication non-adherence was 139 (32.9%). Female gender [AOR = 3.29, 95% CI (2.04, 5.31)], illiteracy [AOR = 2.17, 95% CI (1.35, 3.50)], unemployment [AOR = 3.40, 95% CI (2.15, 5.38)], treatment duration greater than 25 months [AOR = 1.89, 95% CI (1.05, 3.41)], and severe ADRs [AOR = 3.94, 95% CI (1.68, 9.23)] were significantly associated with Antidepressant medication non-adherence. Being female, illiterate, unemployed, having a treatment duration of more than 25 months, and experiencing severe adverse drug reactions were significantly associated with non-adherence. These findings highlight the need for targeted interventions to improve adherence among these high-risk groups.

## Introduction

   Major Depressive Disorder (MDD) presents with a combination of physical, emotional, and cognitive symptoms that severely impact an individual’s ability to function. These symptoms are often persistent and can lead to long-term disability if not addressed with proper treatment, including psychotherapy, medication, and lifestyle interventions^1^. According to the World Health Organization (WHO), MDD was ranked as the third leading cause of disease burden worldwide in 2008. It is projected to become the primary cause by 2030^2^. There was a range of 2 to 21% lifetime prevalence of MDD, with certain European countries having the highest rates and some Asian countries having the lowest^3^. While antidepressant medications are the preferred treatment for MDD, the FDA has also approved their use for other psychiatric and medical conditions^4^. The prevalence of major depression disorder among adults in Ethiopia ranges (17.5% − 38.7%)^5,6^.

   Non-adherence encompasses a range of patient behaviors, including missing prescribed doses, skipping or temporarily stopping medication, reducing or increasing the dose without medical advice, failing to initiate or refill prescriptions, and prematurely discontinuing treatment^7–9^. Non adherence is likely to worsen in developing nations, with significant consequences^10,11^. In Ethiopia, previously study indicated non-adherence rates ranged from 26–57.1%,previous suicide attempt, medication side effects, moderate to high self-stigma, poor quality of life, and lifetime alcohol use were factors strongly influencing treatment non adherence^7,12^ .

  Most existing studies on non-adherence to antidepressants have been conducted in high-income countries, with few focusing on Ethiopia, particularly in the Northwest region^12^. Worldwide, Recent large-scale studies in the United States and five European countries found non-adherence rates of 42.9% in the US and 46.2% in Europe among adults prescribed antidepressants^13^. Africa, Data from African countries is limited, but studies suggest that non-adherence rates may be higher than in high-income countries, often exceeding 50% in some settings^7^. Studies in Ethiopia show a wide range of non-adherence rates to antidepressants and other psychotropic medications: A 2019 study at St. Amanuel Mental Specialized Hospital in Addis Ababa found a non-adherence prevalence of 26% among adults with depressive disorders^14^. A 2025 systematic review and meta-analysis found the pooled prevalence of psychotropic medication non-adherence in Ethiopia to be 44% (43.98%, 95% CI 38.15–49.81), with regional variations: Harari: 52.85%, Amhara: 52.4%, Oromia: 46.4%, Addis Ababa: 39.8%, Tigray: 27.4%^15^.Cultural beliefs, stigma, and traditional healing practices in rural or semi-urban regions like Northwest Ethiopia may significantly impact medication adherence but have not been fully explored^16^.

  Non-adherence with antidepressant drug therapy can have negative outcomes such as functional impairment, higher healthcare utilization, relapse, and hospitalization, delays in establishing remission, poor quality of life, premature mortality, substance abuse, and suicide^13,17^. Non-adherence to medicine remains a serious barrier for people living with mental diseases, as well as healthcare providers and the overall healthcare system. This complicated phenomena creates significant barriers to effective treatment, needing a thorough knowledge of the underlying causes and the creation of specific interventions to improve adherence and overall mental health outcomes^18,19^.

Existing studies suggest that inadequate follow-up practices by healthcare providers can lead to poor antidepressant medication non adherence among MDD patients, but there is limited research into how these practices are implemented in Ethiopia’s rural and semi-rural settings^20^.

  In Ethiopia, existing studies report highly variable non-adherence rates, reflecting methodological inconsistencies and regional disparities in healthcare access and cultural factors^14^. Studies in Ethiopia highlighted regional disparities, with non-adherence ranging from 27.4% in Tigray to 52.8% in Harari, underscoring the need for multi-center data to guide localized interventions^15^.This gap in research inspired the current study, which aimed to determine the factors (such as sociodemographic and clinical characteristics, as well as ADR Severity Score and Naranjo Probability Scale) that predict non adherence to antidepressant drugs among major depressive disorder patients at public hospital psychiatric clinics in Ethiopia.

## Method and materials

### Study design and study area

  A hospital-based, multicenter cross-sectional study was conducted from June 12, 2024, to November 13, 2024, across three comprehensive and specialized hospitals (CSHs) in Northwest Ethiopia: Felegehiwot, Debre Tabor, and Gondar. The region is selected due to various socio-cultural experiences are found across the population which makes the study to be generalized. The three CSHs were selected by lottery method out of the total of eight compressive and specialized hospitals found in Amhara regional state. It is 37.5% of the total CSHs which is statistically appropriate according to WHO criteria (> 30%). These at public hospital psychiatric clinics provide both inpatient and outpatient services, including treatment for bipolar disorder, depression, substance misuse, and schizophrenia. Over the study period, the clinics treated approximately 265, 224, and 326 psychiatric patients at Felegehiwot, Debre Tabor, and Gondar hospitals, respectively. Mental health services were delivered by a combined workforce of 3 psychiatrists and 15 psychiatric nurses at Felegehiwot, 5 psychiatrists and 10 psychiatric nurses at Debre Tabor, and 6 psychiatrists and 12 psychiatric nurses at Gondar.

### Population

  All patients with major depressive disorders on follow up at Felegehiowt, Debre Tabor, and Gondar compressive and specialized at public hospital psychiatric clinics were taken as reference population and Patients with major depressive disorders and who have been taking antidepressant for at least one month at Felegehiowt, Debre Tabor, and Gondar compressive and specialized hospitals in psychiatric clinic during the study period were considered as study population.

### Inclusion and exclusion criteria

  The study included patients who were receiving antidepressant drugs for at least one months. Patients or care givers who were unable to converse and had a serious general medical condition (an illness, injury, impairment, or physical or mental condition which requires: Overnight hospitalization, including the period of incapacity or subsequent treatment in connection with the overnight care were excluded.

### Study variables

  Magnitude and factors of non-adherence to antidepressants were our outcome variables whereas antidepressants severity and ADRs, sociodemographic characteristics of the patient including (age, gender, residence, and disease duration) were the independent variable.

### Sample size determination and sampling technique

  The sample size was determined by using the single population proportion formula using EPI Info version 7.2. Using an assumption of 95% level of confidence, 5% marginal error and 26% non-adherence among patients with depressive disorder^14^, and after adding 10% non-responses which gave a total sample size of 568.

$$\:n\:=\frac{{\left({z}_{\frac{a}{2}}\right)}^{2}\times\:p\times\:\left(1-p\right)}{{d}^{2}}=\frac{{\left(1.96\right)}^{2}\times\:0.5\times\:\left(1-0.26\right)}{{\left(0.05\right)}^{2}}+10$$

*n* = 568.

**Fig. 1:**
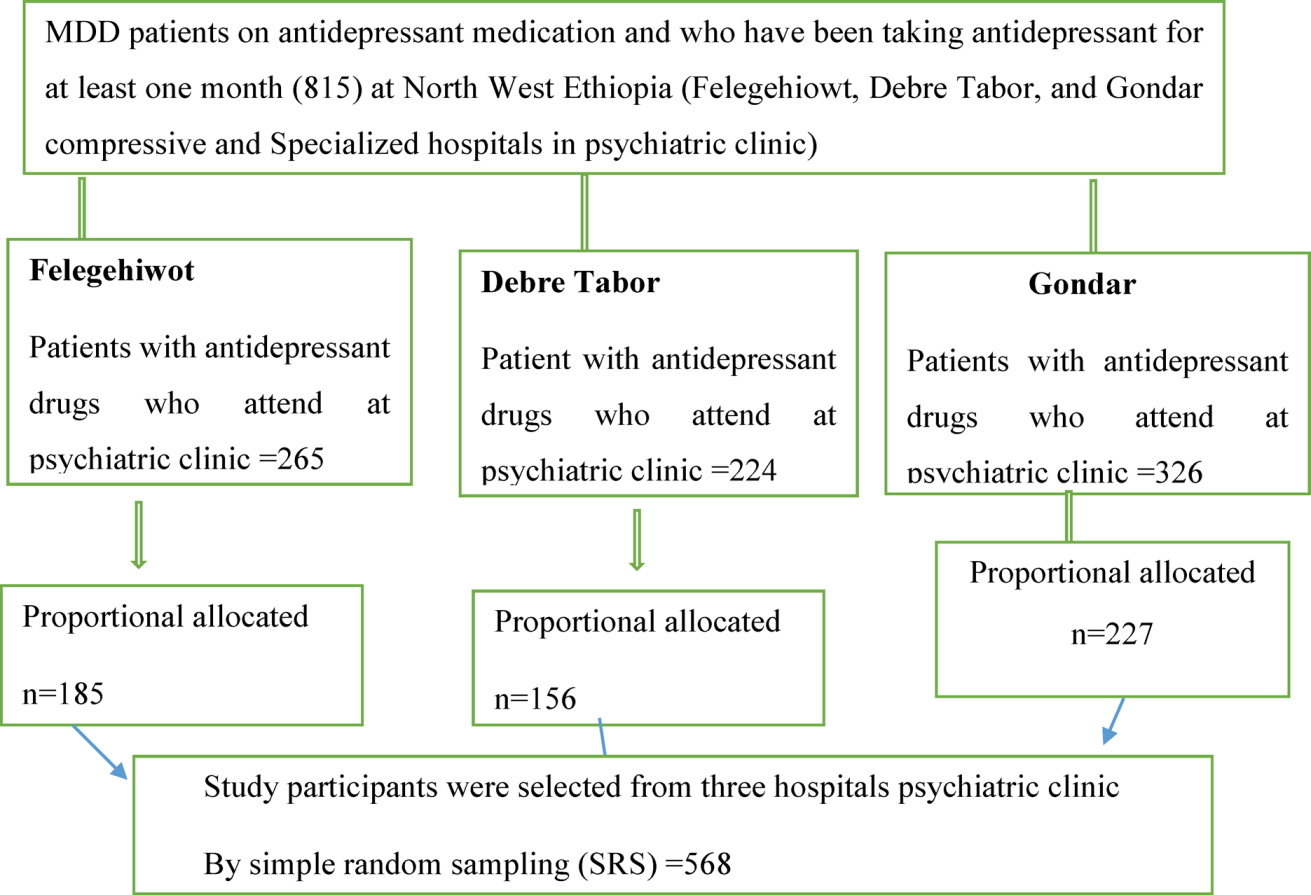
Schematic representation of sampling procedures of MDD patients on antidepressant medication at public hospital psychiatric clinics in Ethiopia, 2024 (*N* = 568).

A systematic random sampling procedure was applied. When patients’ records arrived at the clinic during data collection days, they were reviewed for inclusion criteria. Selecting every nth patient from a list of patients attending the psychiatric clinics during the study period. When a patient was absent when the chart was delivered, another patient who meets the requirements is added. The follow-up patients noted in the record book served as the study’s sampling frame (Fig. 1).

### Data collection procedure

  Questionnaire Data was prepared in English, then translated into the local language, Amharic, and back into English. Primary data was gathered from individual patients. These were constructed questions that included socio-demographic and economic characteristics, clinical-related factors. The Royal College of Psychiatrists established the Antidepressant Side-Effect Checklist (ASEC), which was used to classify as mild, moderate, or severe^21^. The Naranjo ADR probability scale (ten items) was used to evaluate antidepressant-related adverse drug reactions. ADR is classified as definite (scoring ≥ 9), probable (score 5–8), possible (score 1–4), and questionable (score 0)^22^, and the non-adherence to antidepressant medication was assessed using a self-reported tablet count tool with pharmacy refill records available in patients’ charts at the time of the interview. By cross verifying patient reported adherence with pharmacy refill patterns, we minimized the potential impact of recall errors and social desirability bias, thereby strengthening the robustness of our findings. Patients were classified as non-adherent if they consumed less than 80% of the prescribed doses, while adherence was defined as taking 80% or more of the prescribed doses^23^. Nine psychiatry nurses (three at each hospital) collected the data. The primary and co-investigators provided oversight during the data collection period.

### Data quality assurance

  Medication adherence was assessed using a validated and modified version of the medication adherence rating scale (internal consistency Cronbach’s alpha = 0.78)^24^. Other surveys were also developed following a review of English literature. Data were collected in Amharic languages based on the patients’ language fluency. Prior to the data collection phase, comprehensive training was provided to the data collectors (psychiatry nurses) and supervisors to ensure consistency in data collection procedures. Before beginning data collecting, a pre-test of questionnaires was conducted on 10% of depression patients receiving antidepressant medication at Dessie Hospital psychiatric clinic to prevent information cross-contamination, as well as to ensure the questionnaire’s linguistic clarity and consistency. Following the pre-test, the questionnaire’s ambiguous terms were corrected. Before the patients left the psychiatric clinic, the questions were reviewed for completeness and revised. On site supervision was conducted by the primary and co-investigators during the data collection period to monitor the process and ensure that protocols were being followed. This included direct observation of interviews and review of completed questionnaires for any errors or missing information. To ensure consistency and correctness, the obtained data was entered into a computer using a double data entry technique.

### Data processing and analysis

  The data was cleaned for completeness, coded, entered into EpiData, transferred, and analyzed with the Statistical Package for Social Science (SPSS) version 26.00. To ensure data accuracy and reduce errors during data entry, we used a double data entry technique, where the data was entered into the system twice by different individuals, and discrepancies were resolved through cross-checking. The descriptive analysis was summarized using frequency and percentage. Bivariable analysis was used to investigate the relationship between the independent and outcome variables.

Variables with a *p*-value less than 0.25 in the bivariable analysis were included for multivariable analysis. To determine significant variables in the model, the variables were first filtered using the backward LR, and then the remaining variables were processed using the entry technique.

 The Hosmer-Lemeshow test was used to assess model fitness. Following correction, an independent variable with a *p*-value of less than or equal to 0.05 was considered significantly linked with the dependent variable. It was presented with an adjusted odds ratio and a 95% confidence interval.

### Ethical consideration

  Ethical approval was obtained from School of Pharmacy ethical approval Review Committee May 22, 2024, SOP (088/2024), College of Medicine and Health Sciences, University of Gondar, Ethiopia. All respondents provided informed consent for participation in the survey. Written and verbal informed consent was obtained from each study participant to confirm their willingness to participate. All participants were given the option to withdraw from the study at any time if they chose not to continue. Confidentiality was ensured by removing any identifying information and securely storing the questionnaires in a locked area after data collection. All methods were carried out following relevant guidelines and regulations based on the Helsinki Legislation.

### Operational definition

  Antidepressants medication are used for the managing of MDD involves a combination of pharmacologic approaches. Tricyclic antidepressants with selective serotonin reuptake (SSRIs) and serotonin norepinephrine reuptake inhibitor (SNRIs) medications are the commonly prescribed pharmacologic drugs^25^.

  Medication adherence was measured by using a self-reported tablet count tool: This method directly measures adherence by quantifying the number of remaining pills. Patients were classified as non-adherent if they consumed less than 80% of the prescribed doses, while adherence was defined as taking 80% or more of the prescribed doses. The adherence percentage was calculated as follows: Adherence (%)=(Total prescribed doses per month − Missed doses per month)divided Total prescribed doses per month)×100^23^.

## Results

### Sociodemographic characteristics of patients

  Four hundred twenty-two patients participated in the study giving a 75% response rate. The mean age of the participants was (43.2 ± 11.49). More than half of the respondents were females 228 (54%). Nearly 214, (50.7%) of them had no complete primary, secondary, higher education. The majority of the patients were not employed 226(53.6%). Approximately 258 patients (61.4%) were from rural areas.179 patients (42.6%) had co-occurring illnesses connected to psychiatry. The mean duration since starting the antidepressant medication was 15.2 ± 9.48 months (Table 1).

**Table 1:** Sociodemographic characteristics of MDD patients on antidepressant medication at public hospital psychiatric clinics in Ethiopia, 2024 (*n* = 422).

Variables	Category	Frequency	Percept
Sex	Male	194	46.0
	Female	228	54.0
Age	(Mean ± standard deviation)	43.2 ± 11.49	
	20–37 years	144	34.1
	38–46 years	129	30.6
	47–54 years	71	16.8
	55–75years	76	18.0
Marital status	Single	48	11.4
	Married	318	75.4
	Divorced	35	8.3
	Widowed	21	5.0
Education	Literate	208	49.3
	Illiterate	214	50.7
Employment	Employed	196	46.4
	Unemployed	226	53.6
Residency	Rural	258	61.4
	Urban	164	38.6
Economic income monthly (ET Birr)	< 620	120	28.4
	630–760	96	22.7
	780–1220	102	24.2
	1230–6000	104	24.6

### Clinical characteristics of the study participants

  Among study participants 179(42.6%) patients had comorbid psychiatric condition. 148(31.5%) participants had moderate level of depression the mean duration since starting the antidepressant drugs was 15.2 ± 9.48 months (Table 2).

**Table 2:** Clinical characteristics of MDD patients on antidepressant medication at public hospital psychiatric clinics in Ethiopia, 2024 (*n* = 422).

Variables	Category	Frequency *n* (%)
Episodes	First	170 (40.6)
	Multiple (≥ 2)	250 (59.1)
Severity of depression	Mild	104 (24.6)
	Moderate	148 (35.1)
	Severe	124 (29.4)
	Absent	46 (10.9)
Comorbid medical condition	Yes**	106 (25.4)
	No	314 (74.6)
Suicidal addition	Yes	124 (29.6)
	No	296 (70.4.6)
Other psychiatry disorders	yes*	179 (42.6)
	No	241 (57.4)
Duration since starting the drugs (months)	6–12	137 (32.5)
	13–24	155 (36.7)
	> 25	130 (30.8)
Medication adherence	Adherence	283 (67.1)
	Non adherence	139 (32.9)

Yes *= bipolar I and II, psychotic features, substance use disorders, schizophrenia, Yes **= hypertension, thyroid toxicities, migraine headache,

### ADR severity score and Naranjo probability scale of study participants

  According to ASEC, the majority of adverse drug reactions (ADRs) were classified as moderate 178 patients (42.2%), followed by mild 132 patients (31.3%) and severe 112 patients (26.5%). According to the Naranjo score, approximately 218 (51.7%) ADRs were possible and 112 (26.5%) were possible (Table 3).

**Table 3:** Severity and probability of antidepressant side effects of MDD patients on antidepressant medication at public hospital psychiatric clinics in Ethiopia, 2024 (*n* = 422).

Variable	Category	*N* (%)
ADR severity	Mild	132 (31.3)
	Moderate	178 (42.2)
	Severe	112 (26.5)
Naranjo probability scale	Absent	92 (21.8)
	Possible	218 (51.7)
	Probable	112 (26.5)

### Determinant factors associated with antidepressant medication non adherence

  Prevalence of non-adherence to antidepressant medication was 32.9% (95 CI; 28.2–37.7%) of patients with depressive disorder were non-adherent to antidepressant treatment. According to bivariate analysis, sex, age, education, residency employment status, other psychiatric illness, duration since starting of drugs (year), severity of ADR and probability of ADR were candidate variables for the final model and entered into multivariable logistic regression. In the multivariate analysis; being female [AOR = 3.29, 95% CI (2.04,5.31)], being have illiterate [AOR = 2.17, 95% CI (1.35,3.50)], unemployed [AOR = 3.40, 95% CI (2.15,5.38)], more than 25 months duration since starting of drugs [AOR = 1.89,95% CI (1.05,3.41)], and severe ADR [AOR = 3.94(1.68–9.23) ] and were significantly associated with antidepressant medication non adherence (Table 4).

**Table 4:** Determinant with antidepressant medication non adherence of MDD patients at public hospital psychiatric clinics in Ethiopia, 2024 (*n* = 422).

Variable	Category	Medication adherence		COR (95%CI)	AOR (95%CI)
		Non adherence	Adherence		
Sex	Female	106 (46.5)	122 (53.5)	2.563 (1.653–3.976)	3.29(2.038–5.31) **
	Male	146 (75.6)	48 (24.7)	1	1
Education	Illiterate	103 (48.1)	111 (51.9)	3.501 (2.307–5.313)	2.17(1.35–3.50) **
	Literate	103 (49.6)	105 (50.4)	1	1
Residency	Rural	183 (70.9)	75 (29.1)	3.359 (2.229–5.063)	1.22(0.77–1.94)
	Urban	69 (42.1)	95 (57.9)	1	1
Employment status	Employed	83 (42.3)	113 (57.7)	1	1
	Unemployed	169 (74.8)	57 (25.2)	4.037 (2.671–6.099)	3.40(2.15–5.38) **
Psychiatric illness	Yes	92(51.4)	87(48.6)	2.237 (1.502–3.331)	1.91(0.95–3.82)
	No	165(67.9)	78(32.1)	1	1
Duration since the start of drug	6–12 months	96 (70.1)	41 (29.9)	1	1
	13–24 months	85 (54.8)	70 (45.2)	1.928 (1.189–3.127)	1.92(0.98–3.34)
	≥ 25 months	71 (54.6)	59 (45.4)	1.946 (1.177–3.217)	1.89(1.05–3.41) *
Severity of ADR	Moderate	160 (87.6)	18(12.2)	1	1
	Severe	82 (73.2)	30 (26.8)	6.00 (2.591–9.893)	3.94(1.68–9.23) *
Probability of ADR	Possible	185 (84.9)	33 (15.1)	1	1
	probable	94 (84.2)	18 (15.8)	2.978 (1.486–5.968)	3.29(0.89–7.24)

Hosmer and Lemeshow goodness of fit *p* = 0.741, *p < 0.05 and **p < 0.01, COR, crude odds ratio; AOR, adjusted odds ratio.

## Discussion

  Depression is a prevalent mental health condition that necessitates appropriate medical intervention. The combination of antidepressant medication and psychotherapy is anticipated to alleviate symptoms^26^.

  This study aimed to evaluate the prevalence of non-adherence to antidepressant medication and identify associated factors among patients with major depressive disorder. Our findings revealed that 32.9% (95% CI 28.2–37.7%) of patients with depressive disorder were non-adherent to their prescribed antidepressant treatment. This prevalence aligns closely with several international studies, including those conducted in Spain (30.1%)^27^, United Kingdom (30% )^28^, USA (28% )^29^ and Asella Ethiopia (37.7%)^7^.

  However, our observed non-adherence rate is lower than reported in studies from Nigeria (44.3% )^11^, Saudi Arabia (52.9% )^30^, India (66.9% )^31^, Japan (63.1% )^32^, Thailand (59% )^33^ and Ethiopia (38.7-50.48%)^6,34^. These variations can be attributed to differences in patient populations, study methodologies, cultural contexts, healthcare system structures, and measurement tools used to assess adherence. Understanding these discrepancies is critical for tailoring interventions that effectively address local barriers to adherence and meet patient-specific needs.

  Conversely, our prevalence is higher than that reported in a prior Ethiopian study which found a 26% non-adherence rate^14^. The disparity may stem from differences in study settings and participant characteristics. The previous study was conducted in a single center and may have included patients with more heterogeneous living conditions, potentially influencing adherence behaviors^35^.

  Overall, these findings underscore the complexity of antidepressant adherence, influenced by multifaceted sociodemographic, clinical, and systemic factors^18,36^. Future research should continue to explore these variables within diverse contexts to inform culturally sensitive and contextually appropriate adherence-enhancement strategies.

  Concerning to the determinant of non-adherence the following variables were identified in our study. Being females, illiterate, unemployed, more than 25months medication follow up, and severe form of ADR were all significant predictors of antidepressant treatment non adherence.

  In this study, being females had 3.29 times higher odds of non-adherence compared to males. This result is consistent with studies from Nepal and Canada, which also indicated that being female was a significant factor for non-adherence to antidepressant medication among patients with major depressive disorder^37,38^. The multiple roles and responsibilities that females often have in Ethiopian society can indeed pose significant challenges for them to adhere to hospital appointments and medication schedules. Balancing the demands of being homemakers, wives, mothers, professionals, and caregivers can lead to time constraints and competing priorities, making it harder for women to prioritize their own health needs^39^. This can impact their ability to attend hospital appointments and adhere to prescribed medication regimens, including antidepressants. Understanding and addressing these social and cultural factors is important in developing effective strategies to support medication adherence among women in Ethiopia. In contrast to this study, men’s in the USA and Belgium were more likely to antidepressant treatment non adherence^40,41^. It suggests that the factors influencing medication adherence can vary significantly across different cultural and societal contexts. While women in Ethiopia may face challenges related to their multiple roles and responsibilities, leading to potential non-adherence to antidepressant treatment, men in the USA and Belgium may have different reasons for non-adherence. This could be related to factors such as stigma around mental health, perceptions of masculinity, or other social and cultural influences that affect men’s attitudes towards seeking and adhering to mental health treatment. Understanding these differences is important for developing targeted interventions to improve medication adherence across diverse populations. It highlights the need for culturally sensitive approaches that take into account the specific challenges and barriers faced by different groups when it comes to managing mental health conditions and adhering to treatment plans.

  The finding that illiterate patients were 2.17 times more likely to be non-adherent to antidepressant medication compared to literate individuals highlights the impact of education and health literacy on medication adherence. This trend is consistent with similar findings in other countries such as Nepal and Spain^27,37^. The potential reasons for this could be multifaceted. Illiterate patients may have limited knowledge about their medical conditions and the prescribed medications, leading to fear of potential side effects. This lack of understanding may contribute to their reluctance to engage in discussions with healthcare professionals about their concerns and the treatment plan. Additionally, illiterate individuals may face barriers in accessing and understanding health information, which can further exacerbate their apprehension about medication adherence. This underscores the importance of tailored educational interventions and communication strategies that are sensitive to the needs of individuals with lower literacy levels. Healthcare providers should strive to employ clear, simple language and visual aids to convey essential information about medications and their potential side effects. Moreover, fostering an open and supportive dialogue with illiterate patients can help address their fears and empower them to actively participate in their treatment decisions, ultimately promoting better adherence to antidepressant medications.

  The finding that unemployed individuals had 3.40 times higher risk of non-adherence compared to employed individuals underscores the impact of socioeconomic factors on medication adherence. This association is consistent with the broader understanding that financial constraints can significantly affect access to healthcare services and medication adherence. Unemployment can lead to financial instability, making it challenging for individuals to afford prescription medications, medical appointments, and other healthcare-related expenses^37^. Moreover, the lack of health insurance coverage among unemployed individuals can further exacerbate these financial barriers. The inability to afford long-term medications and frequent follow-up appointments due to limited financial resources may contribute to non-adherence to antidepressant medications and other treatments. Additionally, the stress and anxiety associated with unemployment can also impact mental health and potentially influence medication adherence. Addressing these challenges requires a multi-faceted approach that includes improving access to affordable healthcare services, providing financial assistance programs for medication costs, and offering support services for individuals experiencing unemployment. Healthcare providers should also consider these socioeconomic factors when developing treatment plans and work collaboratively with patients to find viable solutions to mitigate financial barriers to medication adherence.

  Our findings indicate that patients with a follow-up time of 13–24 months and those with over 25 months of follow-up had a higher risk of non-adherence compared to those within the 6–12 months’ timeframe. This change highlights that as the follow-up duration increases, the likelihood of patients becoming non-adherent to treatment also rises. Possible explanations for this trend could include treatment fatigue, loss of motivation, or life circumstances affecting the ability to adhere to long-term treatment plans. Additionally, prolonged use of antidepressants can lead to concerns about tolerance, dependence, and side effects, incontinence and relapse of the underlying disorder, which can impact a patient’s willingness to continue taking the medication and contribute to non-adherence.

  Furthermore, the study’s observation that patients experiencing severe side effects from antidepressant medication had 3.94 times higher odds of non-adherence compared to those with moderate side effects underscores the impact of medication tolerability on adherence. The need to add other medications for managing severe side effects could indeed complicate the treatment regimen and potentially affect adherence. These findings highlight the importance of considering the long-term implications and potential side effects of antidepressant medications when developing treatment plans. Healthcare providers should regularly assess patients for medication tolerance and side effects, as well as provide appropriate support and education to address any concerns related to long-term use. Additionally, personalized treatment approaches that take into account individual patient experiences and challenges with medication adherence can help optimize treatment outcomes.

## Strength and limitation

  Generally, this study provides valuable insights into the prevalence and extent of non-adherence to antidepressant medication, identifying key determinant factors that contribute to this issue. By exploring these factors, the study offers a foundation for improving patient adherence in clinical settings. The data collection was conducted by trained psychiatry nurses who were familiar with the patient population. Collecting data in the native language of participants ensured that questions were understood correctly, minimizing miscommunication. Additionally, nurses were able to offer immediate health information and guidance to non-adherent patients, which may have had a positive impact on care.

  A major limitation is the cross-sectional nature of the study, which restricts the ability to establish a causal relationship between the identified factors and non-adherence. The possibility of bidirectional influences patients may discontinue medication due to ADRs, while non-adherence itself may contribute to withdrawal symptoms or other adverse events that could be misclassified as ADRs. This design only captures a snapshot in time and does not track changes in adherence over the treatment period. The response rate was lower than the sample size determined, which may introduce selection bias and affect the generalizability of the results. Non-responding patients may have different characteristics or reasons for non-adherence that are not captured in the study. Some of the secondary data required for the study were missing from patient charts. This lack of data could limit the completeness and accuracy of the findings, as important clinical information about non-adherence might not have been fully evaluated. Furthermore, we have incorporated a strong recommendation for future longitudinal or prospective cohort studies, which are better suited to disentangle the temporal and causal pathways underlying these complex relationships.

## Conclusion

  This study reveals a considerable proportion of non-adherence to antidepressant medications among patients with major depressive disorder in Ethiopia, representing a critical barrier to effective mental health care delivery. Non-adherence was notably higher among women, individuals with limited literacy, the unemployed, those undergoing prolonged treatment (≥ 25 months), and patients reporting severe adverse drug reactions. These findings highlight the need for nuanced, context-specific strategies to improve treatment adherence. From a clinical standpoint, implementing structured, patient-centered follow-up protocols, enhancing psychoeducation to address health literacy gaps, proactively managing adverse effects, and delivering targeted psychosocial support are essential interventions. At the policy level, efforts should focus on the integration of mental health services within community-based care frameworks, bolstering medication supply chains, and ensuring equitable access to mental health resources. Collectively, these approaches can inform the development of sustainable, evidence-informed strategies to enhance medication adherence and improve clinical outcomes for individuals with depression in low-resource settings.

## Recommendation

  Based on the findings of our study on antidepressant medication non-adherence among patients with MDD, we recommend several strategies for respective organizations, including healthcare institutions, policy makers, and research bodies, to address the challenges identified. Healthcare providers should establish patient education programs to increase awareness about the importance of medication adherence. Governments should work to ensure that antidepressant medications are affordable and accessible, particularly for unemployed or low-income individuals who are at higher risk of non-adherence. More research is needed to develop and test adherence-enhancing interventions that are tailored to the needs of specific populations, such as females, the unemployed, and those with prolonged treatment periods. Non-Governmental Organizations (NGOs) should advocate for improved mental health infrastructure, particularly in low-resource settings like Ethiopia.

## Data Availability

The dataset used and analyzed during this study is available from the corresponding author upon reasonable request.

## Abbreviations

ASEC: Antidepressant side effect checklist
ADR: Adverse drug reaction
CSHs: Compressive and specialized hospitals
MDD: Major depressive disorder
SPSS: Statistical package for social science
SNRI: Serotonin norepinephrine reuptake inhibitor
SSRI: Selective serotonin reuptake
US: United States
WHO: World Health Organization
